# Cannabinoid receptor type-1: breaking the dogmas

**DOI:** 10.12688/f1000research.8245.1

**Published:** 2016-05-24

**Authors:** Arnau Busquets Garcia, Edgar Soria-Gomez, Luigi Bellocchio, Giovanni Marsicano

**Affiliations:** 1 Endocannabinoids and Neuroadaptation, INSERM U1215 NeuroCentre Magendie, Bordeaux, 33077, France; 2 University of Bordeaux, Bordeaux, France

**Keywords:** Endocannabinoid system, allosteric modulator, molecular pharmacology, cannabinoid ligands, CB1 receptor signaling

## Abstract

The endocannabinoid system (ECS) is abundantly expressed in the brain. This system regulates a plethora of physiological functions and is composed of cannabinoid receptors, their endogenous ligands (endocannabinoids), and the enzymes involved in the metabolism of endocannabinoids. In this review, we highlight the new advances in cannabinoid signaling, focusing on a key component of the ECS, the type-1 cannabinoid receptor (CB _1_). In recent years, the development of new imaging and molecular tools has demonstrated that this receptor can be distributed in many cell types (e.g., neuronal or glial cells) and intracellular compartments (e.g., mitochondria). Interestingly, cellular and molecular effects are differentially mediated by CB _1_ receptors according to their specific localization (e.g., glutamatergic or GABAergic neurons). Moreover, this receptor is expressed in the periphery, where it can modulate periphery-brain connections. Finally, the better understanding of the CB _1_ receptor structure led researchers to propose interesting and new allosteric modulators. Thus, the advances and the new directions of the CB _1_ receptor field will provide new insights and better approaches to profit from its interesting therapeutic profile.

## Introduction

The endocannabinoid system (ECS) is composed of G protein-coupled cannabinoid receptors, namely cannabinoid receptor-1 (CB _1_) and cannabinoid receptor-2 (CB _2_) ^1, 2^; the endogenous cannabinoids called endocannabinoids, such as the lipids anandamide and 2-arachidonoylglycerol ^3, 4^; and the enzymes involved in their synthesis and inactivation ^5^. The family of endocannabinoids has recently grown to include a group of peptide ligands (so-called pepcans) and other lipid molecules, such as lipoxin and pregnenolone, interestingly acting as allosteric enhancers or signal-specific inhibitors (SSIs) of CB _1_ receptors ^6^.

One of the main characteristics of the ECS is its broad distribution throughout the body. In this review, we will specifically focus our attention on the CB _1_ receptor-dependent functions in the nervous system (particularly the brain). The CB _1_ receptor is considered the most abundant metabotropic receptor in the brain ^7^. It was cloned in 1990 ^1^ and its distribution has been well characterized in both rodents ^8, 9^ and humans ^10^. These receptors are particularly rich in the central nervous system ^11, 12^, where they control a wide spectrum of physiological and pathological conditions, including brain development, learning and memory, motor behavior, regulation of appetite, body temperature, pain perception, inflammation, and they are involved in various psychiatric, neurological, and neurodevelopmental disorders ^13– 17^.

This review highlights recent findings that challenge or extend accepted “dogmas” of CB _1_ receptor signaling. Thus, it discusses where CB _1_ receptors are localized, the importance of CB _1_ receptors outside the brain, and new strategies to pharmacologically act on these receptors. Importantly, the understanding of where, which, and how CB _1_ receptor function is mandatory to improve the pharmacological strategies to act on this promising therapeutic target.

## Localization of CB _1_ receptors in different neuronal types

CB _1_ receptor localization has been widely studied during the last few decades ^18^. Thus, early studies provided strong evidence for a presynaptic localization of CB _1_ receptors, from where they can control the neurotransmitter release ^7, 19^. However, the somatodendritic localization of CB _1_ receptors cannot be discarded, as processes of self-inhibition through these receptors have been demonstrated in the cortex ^20– 23^. According to this, recent work describes that somatodendritic CB _1_ receptors control a specific postsynaptic signaling cascade important for the cognitive impairment induced by cannabinoids ^24^. Therefore, more studies are needed to clarify the relative involvement of pre- or post-synaptic CB _1_ receptors in brain functions and how this can affect our general view of how the ECS controls synaptic transmission.

Interestingly, new experimental approaches (e.g., imaging tools) have shown the expression of CB _1_ receptors in different neuronal types, including GABAergic, glutamatergic, and serotonergic neurons, among others ^8, 25– 28^. Moreover, although the anatomical presence of CB _1_ receptors in cholinergic, noradrenergic, or dopaminergic neurons has not been fully characterized, cannabinoids are known to control acetylcholine and dopamine release ^29, 30^. For example, it has been recently shown that CB _1_ receptors can specifically control cholinergic over glutamatergic transmission at single synapses that co-release both neurotransmitters ^31^.

Importantly, the expression levels of CB _1_ receptors can drastically differ among different cell types and can diverge between different brain regions ^12, 25, 32, 33^. This widely distributed and differential expression in the brain reflects the complexity, and can explain the variety of functions, of the ECS. For instance, this specific distribution can explain some of the bimodal effects of cannabinoid drugs ^34, 35^. Thus, recent studies demonstrated how CB _1_ receptors localized in GABAergic neurons can control food intake ^34^, running related behaviors ^36, 37^, drug addiction ^38, 39^, and learning and memory processes ^40, 41^, among other behaviors, whereas CB _1_ receptors localized in glutamatergic neurons control neuroprotection ^42^, olfactory processes ^25^, fear memories ^43^, social behaviors ^44^, and anxiety ^35^, among others. Moreover, CB _1_ receptors present in serotonergic neurons can modulate emotional responses ^45^.

## Localization of CB _1_ receptors in other cell types or intracellular organelles

The biased neuron-centric view in the ECS field changed when CB _1_ receptors were found in another type of brain cells, the glial cells ^46– 49^. Moreover, recent studies have demonstrated how the astroglial CB _1_ receptor can modulate important physiological functions in behavior and synaptic plasticity such as learning and memory and long-term depression in the hippocampus ^50– 52^. Therefore, this receptor can shape synaptic transmission via astroglial signaling ^53^. By doing this, it modulates the effects of exogenous cannabinoids on working memory ^46^ and, notably, can also determine the selective activity of specific circuits in the striatum ^54^. Thus, the improvement of the current tools will consolidate this knowledge to better elucidate the role of CB _1_ receptors and astrocytes on brain functioning ^55^. Interestingly, recent findings have shown how CB _1_ receptors can modulate microglia activation, suggesting its presence in this cell type ^49^.

Although CB _1_ receptors are localized primarily at the plasma membrane, more and more evidence suggests the presence of functional intracellular CB _1_ receptors ^56, 57^. For instance, a portion of these receptors is functionally present in cell mitochondria ^58^. In the past, previous data showed that cannabinoids can alter mitochondrial functions, but these effects were fully ascribed to unspecific membrane disturbance induced by these lipid molecules ^59, 60^. However, recent results challenge this idea, indicating that CB _1_ receptors are also present in mitochondrial membranes in the periphery, such as in spermatozoa ^61^ or skeletal muscles ^62^, and in the brain, where they directly regulate mitochondrial oxidative phosphorylation (OXPHOS) activity ^58, 63, 64^ or can impact feeding behavior ^65^. However, further studies and more direct, specific, and powerful tools are needed to investigate the role of mitochondrial or other intracellular CB _1_ receptors on synaptic transmission, brain functions, and behavior. Interestingly, brain mitochondrial functions have been recently causally associated to anxiety-related responses in the nucleus accumbens ^66^, demonstrating how brain energetics can impact behavior.

## Localization of CB _1_ receptors in the periphery

In the last two decades, CB _1_ receptors have been described in a number of peripheral tissues, including fat tissue ^67^, gastrointestinal tract ^68^, mouth and oral cavity ^69^, eye ^70^, cardiovascular system ^71^, liver ^72^, pancreas ^73^, immune system ^74^, bone ^75^, skin ^76^, and skeletal muscle ^77^. Indeed, it seems that the ECS is present in a large majority of tissues and its specific functions have recently been investigated ^78^.

The complex interactions between peripheral organs and the central nervous system raised a particular interest within the neuroscience field. In this sense, it is worth discussing how the peripheral processes modulated by the CB _1_ receptors are affecting the central nervous system functions. A recent study demonstrated that the peripheral sympathetic activity controlled by CB _1_ receptors is necessary for central functions, such as hypophagia and anxiety-like effects ^79^. Other potential examples of the roles of CB _1_ receptors in the periphery-brain connection are the control of the release of stress hormones from the adrenal glands ^80^ or the modulation of gut functions impacting on behavioral responses. Indeed, a close interaction between adipose tissue, gut bacteria, and the endocannabinoid system has been proposed in the context of obesity ^81, 82^.

## New advances in the CB _1_ receptor pharmacology

Several orthosteric ligands of CB _1_ receptors have been described in the last few decades, including natural or synthetic CB _1_ receptor agonists (e.g., Δ ^9^-tetrahydrocannabinol [THC], CP-55,940), antagonists (e.g., rimonabant), and orthosteric endocannabinoids ^6, 83^. Moreover, endocannabinoids seem also to target non-cannabinoid receptors (e.g., G protein-coupled receptor 55 receptors) ^84, 85^ and ion channels (e.g., serotonergic, nicotinic acetylcholine receptors, or vanilloid receptors) ^86^, particularly at concentrations at which they have been found to interact with CB _1_ or CB _2_ receptors ^6, 87^. Notably, the orthosteric action of CB _1_ receptor agonists and antagonists induces important side effects ^88, 89^. For example, rimonabant, known as a partial antagonist/inverse agonist, showed different side effects in humans ^88^. In this sense, different strategies have been shown to improve the safety profile and overcome the side effects induced by CB _1_ antagonists, such as the neutral CB _1_ antagonists ^90^.

Interestingly, the pharmacology of CB _1_ receptors is nowadays also focused in the recent developments on putative allosteric binding sites of these receptors and how this can be translated into new therapeutic approaches. As cannabinoid ligands present an interesting therapeutic profile ^91^, the development of new and safer drugs such as CB _1_ receptor allosteric modulators is needed. Indeed, this strategy has become a hot topic in the G protein-coupled receptors field and there are different positive and negative allosteric modulators described (PAMs and NAMs, respectively) ^92, 93^. Consequently, different compounds have been developed as exogenous CB _1_ allosteric modulators, including the indole derivatives (e.g., the NAM “ORG” compounds) ^94^, urea derivatives (e.g., the NAM PSNCBAM-1) ^95^, and other small molecules that also display a PAM profile, such as RTI-371 ^96^. Importantly, recent work also identified natural PAMs and NAMs of CB _1_ receptors, such as the lipoxin A4, the hemopressin pepcan-12, and pregnenolone ^97, 98^, which might represent model chemical structures for the development of new drugs. Although numerous studies have fully characterized the chemical and signaling properties of these new synthetic or natural compounds ^97, 98^, the *in vivo* effects of all these drugs modulating physiological or pathological conditions constitutes an emerging area in the cannabinoid field. In this context, the neurosteroid pregnenolone exerts peculiar effects on CB _1_ receptor signaling. Indeed, pregnenolone, by binding to a specific identified site on CB _1_ receptors, displays an interesting SSI profile: whereas CB _1_-dependent modulation of cytoplasmic cyclic AMP signaling is unaltered by pregnenolone, the neurosteroid fully blocks the activation of extracellularly regulated kinases (ERKs) and the inhibition of mitochondrial activity by cannabinoids ^63^. By these mechanisms, the SSI pregnenolone blocks different central effects of THC, including memory impairment, hypolocomotion, and cannabinoid self-administration in rodents ^63^. Other compounds have been shown to alter CB _1_ receptor-dependent effects. For instance, the synthetic PAM ZCZ011 reduces neuropathic pain ^99^, whereas the PAM lipoxin A4 shows anti-inflammatory effects ^100^. Interestingly, it was recently shown that cannabidiol, which has been previously reported as a CB _1_ receptor antagonist, behaves also as a non-competitive NAM of CB _1_ receptors, despite its low affinity to these receptors ^101^.

The allosteric modulators of CB _1_ receptors are not the only therapeutic agents recently proposed. Indeed, the effects of several phytocannabinoids in preclinical models of central nervous system diseases and, where available, clinical trials have been investigated, suggesting a promising phytocannabinoid-based medicine ^102^. Another factor that can change the CB _1_ receptor pharmacology is heteromerization with other receptors. Heteromers of CB _1_ receptors and other proteins recently emerged as an important target of the *in vivo* effects of cannabinoids ^103– 105^. Notably, these heterocomplexes could be potentially modulated ^104^ and this implies another pharmacological tool to act on CB _1_ receptor signaling. Moreover, present evidence points to the membrane environment as another critical regulator of CB _1_ receptor signaling, and this can be potentially exploited for the development of novel therapeutic compounds ^106^. Finally, a G protein-coupled receptor such as the CB _1_ receptor may also have a constitutive, ligand-free mode of signaling, as has been shown in hippocampal GABAergic synapses ^107^. All of these new ideas demonstrate that the research community may dedicate more effort to tackle CB _1_ receptors.

## Conclusions

**Figure 1.**
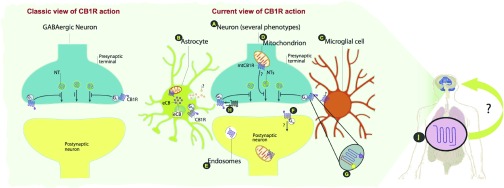
Schematic comparison between the classic and the current view of the CB _1_ receptor functional expression. On the left panel, the classic view of the CB _1_ receptor is represented. The CB _1_ receptor was thought to be exclusively localized in GABAergic neurons, where it was demonstrated to inhibit neurotransmitter release. On the right panel, the current view of the CB _1_ receptor is illustrated. Different advances have completely changed this picture: ( **A**) The CB _1_ receptor is present in different neuronal types and in glial cells, both in astrocytes ( **B**) and potentially in microglia ( **C**). Furthermore, it is found intracellularly in the mitochondria ( **D**) and endosomes ( **E**). The view of a canonical retrograde system changed after the CB _1_ receptor localization in postsynaptic somatodentritic neurons was demonstrated ( **F**). Nowadays, we know that CB _1_ receptor presents allosteric binding sites ( **G**) and that it could form heteromers ( **H**). Beyond the brain, the CB _1_ receptor is widely expressed in the periphery ( **I**), where it can modulate the periphery-brain connection. All of this new knowledge reflects the complexity of the central nervous system and the advance in neuroscience, positing the CB _1_ receptor as an ideal tool for studying brain functions. CB _1_, cannabinoid receptor-1; CB _2_, cannabinoid receptor-2; eCB, endocannabinoid; NT, neurotransmitter.

This short review focused on the new findings in CB _1_ receptor research. However, the ECS comprises other components such as CB _2_ receptors, the endocannabinoids, and the enzymes responsible for their synthesis and degradation. In this sense, recent advances have demonstrated the importance of CB _2_ receptors in the brain ^108– 110^, the presence of other endocannabinoid-like molecules ^111, 112^, other potential receptors that can be activated by endocannabinoids ^87^, and interesting findings regarding the localization and pharmacology of the enzymes involved in the metabolism of these endocannabinoids ^113, 114^. In brief, the actual picture of how the endocannabinoid system works is quite complicated and more efforts are needed to try to merge the old and the new ideas in this field ( Figure 1).

An open question in the cannabinoid field is whether the cellular diversity of CB _1_ functions could improve the therapeutic exploitation of cannabinoid-based drugs. One can speculate whether different CB _1 _ligands can mediate different signaling pathways by selectively controlling different CB _1_ receptors present in different cellular populations. Likewise, it is possible that specific drugs could target exclusively mitochondrial CB _1_ (mtCB _1_) receptors or could avoid activation of intracellular pools of CB _1_. More studies will be needed to answer these questions, but there is already some evidence demonstrating a different pharmacological profile between CB _1_ receptors expressed in GABAergic and glutamatergic cells. Thus, “glutamatergic” CB _1_ receptors are more sensitive to low doses of agonists and are endowed with stronger intracellular coupling, whereas “GABAergic” pools of the receptor are activated by higher doses of agonists and produce lower activation of G proteins ^34, 35, 43, 115^. Therefore, one could speculate that specific compounds able to selectively activate different cellular subpopulations of CB _1_ receptors could be developed. Moreover, combinations of drugs able to modulate glutamatergic or GABAergic neurotransmission with cannabinoid agonists have been shown to promote specific effects of CB _1_ receptors and inhibit others ^116^. It is also interesting to note that both perisomatic and dendritic GABAergic synapses use phasic endocannabinoid signaling, but the tonic form of cannabinoid signaling is present only in perisomatic cells ^107^. Moreover, a recent study ^80^ shows that the peptide endocannabinoids, known as pepcans, act as endogenous allosteric modulators of CB _1_ activity exclusively on noradrenergic neurons **,** demonstrating a cell type-specific regulatory role on endocannabinoid signaling. All of these new and exciting findings suggest that the better we understand cannabinoid signaling, the closer we are to developing specific and local pharmacological drugs that may have importance in brain disorders.

Overall, the new and exciting findings suggesting different and specific localizations of the ECS components and the new strategies proposed to tackle their activity of this receptor open the door to new questions ( Table 1). Indeed, the endocannabinoid system has been related to many physiological and pathological functions ^13, 18, 117^, and the better understanding of these new evidences will bring more light to exploit the therapeutically beneficial properties of this widely spread neuromodulator system in the brain and in the body.

**Table 1.** Open questions in the cannabinoid receptor-1 (CB _1_) receptor field.

Open questions in the endocannabinoid field.
Is the cell type-specific CB _1_ receptor signaling an open door to develop new therapeutic tools?
Is the endocannabinoid system exclusively a retrograde neuromodulator system?
How is the subcellular CB _1_ receptor distributed in the different cell types?
How can CB _1_ receptors control neurotransmitter co-release?
Which physiological and pathological functions are modulated by intracellular CB _1_ receptors?
Is there specific or differential CB _2_ receptor expression in different cell types?
Is the allosteric modulation of CB _1_ receptors a good therapeutic approach for pathological conditions?
Will it be possible to create compounds that target CB _1_ receptors in specific cell types or subcellular localizations?

CB _1_, cannabinoid receptor-1; CB _2_, cannabinoid receptor-2.

## Abbreviations

CB _1_, cannabinoid receptor-1; CB _2_, cannabinoid receptor-2; ECS, endocannabinoid system; NAM, negative allosteric modulator; PAM, positive allosteric modulator; SSI, signal-specific inhibitor; THC, Δ9-tetrahydrocannabinol.
